# Multifactorial etiology of progressive supranuclear palsy (PSP): the genetic component

**DOI:** 10.1007/s00401-025-02898-z

**Published:** 2025-06-04

**Authors:** Ulrich Müller, Günter Höglinger, Dennis W. Dickson

**Affiliations:** 1 Institute of Human Genetics, Justus-Liebig-University, Schlangenzahl 14, 35392 Giessen, Germany; 2 Center for Human Genetics, MVZ diagnosticum, Altenhöferallee 3, 60438 Frankfurt, Germany; 3 Department of Neurology and German Center for Neurodegenerative Diseases, Ludwig-Maximilians-University, Marchioninistr. 15, Munich, 81377 Germany; 4 Mayo Clinic, 4500 San Pablo Road, 32224 Jacksonville, Florida United States of America

## Abstract

Progressive supranuclear palsy (PSP) is mainly a sporadic disease. It has a multifactorial etiology and an interaction between environmental and genetic factors causes disease. While elucidation of environmental risks for PSP is still in its infancy, much has been learned about the genetic etiological component of PSP during the past few years. This article reviews genes that convey risk for PSP. All genes have been identified in association studies. Only those genes with the standard threshold for genome-wide significance of *P* < 5E-8 are covered. These genes include *MAPT, KANSL1, PLEKHM1, STX6, MOBP, EIF2AK3, SLC01 A2, DUSP10, APOE, RUNX2, TRIM11, NFASC/CNTN2* and *LRRK2.* The physiologic function of these genes is described and their potential role in the etiology of PSP is discussed.

## Clinical findings in and neuropathology of progressive supranuclear paly (PSP)

The atypical Parkinson syndrome progressive supranuclear palsy (PSP) belongs to the group of tauopathies. It occurs at a prevalence of about 5–6/100,000 (range 1–25/100,000) [60]. Age at onset most commonly lies between the mid- and late- 60 s. The main clinical symptoms of typical PSP (Steele-Richardson-Olszewski syndrome) include difficulties walking and loss of balance with frequent falls, dysarthria, dysphagia, frontal dementia, and—most characteristically—vertical supranuclear gaze palsy mainly affecting downward eye movements [1, 42, 60, 80].

Several additional symptoms can occur that characterize various subtypes of PSP. In PSP-P Parkinsonian symptoms are pronounced such as rigidity, tremor, and limb bradykinesia; in PSP-F frontal cognitive symptoms are most prominent; postural instability is severe in PSP-PI; additional features in PSP-SL affect speech and language. Further subtypes include PSP-CBS (corticobasal syndrome with cortical sensory loss, limb rigidity and apraxia), PSP-PGF (most prominent affection of gait failure and freezing), PSP-OM (mainly ocular motor dysfunction) [20, 54].

Neurodegenerative changes have been found in various brain regions of PSP patients. Most commonly affected areas include the cerebral cortex (mainly motor cortex), basal ganglia (mainly caudate nucleus and substantia nigra), hypothalamus and subthalamic nucleus, midbrain (especially the tectum and oculomotor nucleus), and the pons (both the tegmentum and the pontine base). The medulla and cerebellum are also usually affected. Neurodegeneration is associated with accumulation of abnormal tau protein. Tau is posttranslational modified (pathologic phosphorylation, acetylation) [16, 95] and misfolded. In PSP cytoplasmic tau aggregates are found in neurons, oligodendroglia, and astrocytes. These accumulations include neurofibrillary tangles in neurons [83], tufted astrocytes [111] and coiled bodies in oligodendroglia [17].

## PSP, a multifactorial disease

PSP is usually a sporadic disease with a multifactorial etiology (i.e., the interaction between environmental and genetic factors). There is evidence that drinking of well water over long periods of time is an environmental risk factor possibly owing to toxic substances such as pesticides [59]. This is supported by an increased risk for PSP in persons living in rural areas who are exposed to pesticides over many years. On the other hand Vidal et al. [100] did not find any environmental risk factors for PSP. Clearly, more studies are required for the identification of novel environmental factors and proof of their validity in the etiology of PSP. The genetic component of risk, however, has been extensively investigated during the last few years. These genetic studies have greatly improved our understanding of pathologic mechanisms in PSP.

## Genes increasing risk for PSP

Several genes that potentially increase risk for PSP have been detected from association studies (Table 1). Genes found by associations are listed and their function is discussed if their association reached genome-wide significance.

**Table 1:** Genes conveying risk for PSP

ne	Location	Gene product	Expression cell type, tissue, organ	Assoc. ref. SNP(s)	P value	References
*MAPT*	17q21.31	tau	Brain: region-specific dysregulation upregulation in frontal cortex	rs 8,070,723 rs 242,557 rs 8,070,723 rs 242,557 rs9468	1.5E-116 4.2E-70 5.57E-144 3.78E-85 1.94E-110	[43] [81] [26]
*KANSL1*	17q21.31	NSL1	Brain-region -specific dysregulation, upregulation in several brain structures	rs242557	4.2E-70	[43]
*PLEKHM1*	17q21.31	PLEKHM1	Brain-region -specific dysregulation, upregulation in several brain structures	rs242557	4.2E-70	[43]
*STX6*	1q25.3	Syntaxin6	Reduced	rs 1,411,478 rs2411478	2.3E-10 7.17E-13	[43] [81]
*MOBP*	3p22,1	MOBP	Both increased and reduced expression were shown in brain	rs1768208 rs1768208	1.0E-16 3.48E-26	[43] [81]
*EIF2AK3*	2p11.2	PERK	decreased	rs7571971 rs7571971	3.2E-13 2.76E-13	[43] [81]
*SLCO1A2*	12p12.1	OATP1A2	Decreased frontal cortex	rs11568563	5.26E-10	[81]
*DUSP10*	1q41	DUSP10	?	rs6687758	1.14E-08	[81]
*APOE*	19q13.31	APOE	?	rs429358 and rs7412	9.57E-16	[101]
*RUNX2*	6p21.32	RUNX2	Increased in brain (microglia and oligodendrocytes)	rs35740963 rs12197948	1.8E-8 5.43E-12	[15] [26]
*TRIM11*	1q42.13	TRIM11	Reduced expression in brain?	rs564309	1.7E-9	[50]
*NFASC*	1q32.1	Neurofascin	Upregulated?	rs12744678	4.15E-8	[39]
*CNTN2*	1q32.1	Contactin-2	Upregulated?	rs12744678	4.15E-8	[39]
*LRRK2*	12q12	Dardarin	Upregulated	rs2242367	1.3E-10	[49]

Genes conveying risk for PSP. References (Ref.) are not given for all association studies. Results of ref. [37] are derived from meta-analyses of several PSP cohorts. References relating to regulation of the various genes are given in the text

(*P* < 5E-8). For any gene that conveys risk we first describe the gene and its function and subsequently report and appraise observations of its risk for PSP.

Before going into the detailed findings of gene associations we want to point out a few caveats of gene assignments by association with SNPs. For example SNPs in high linkage disequilibrium regions may tag multiple genes and the assignment of the “correct” gene might be difficult [28, 97]. Furthermore SNPs are usually located in non-coding regions and might affect distant regulatory regions rather than the nearest gene [28, 97]. This said we describe the findings.

***MAPT*** encodes microtubule-associated protein tau and is highly expressed in neurons and to a lesser extent in oligodendrocytes. The *MAPT* gene is located in 17q21.31 (nucleotide positions: 45,894,527–46,028,334) and is composed of 16 exons. Exons 2,3,10 are alternatively spliced leading to six isoforms of tau [7]. Exons 9–12 contain four repeat sequences that code for the microtubule binding domains of tau. These domains are composed of imperfect repeats of 31 or 32 amino acids (four highly conserved blocks of 18 amino acids, separated by either 13 or 14 different amino acid residues) in the carboxyl terminus of the protein [34, 95]. Exclusion of exon 10 results in tau proteins containing 3 imperfect repeats (3R, −10) and inclusion of exon 10 gives rise to tau proteins containing 4 repeats (4R, + 10). There are two major haplotypes, H1 and H2, spanning approximately 1.8 Mb which contain the entire *MAPT* gene. H2 is defined by an inversion of approx. 900 kb relative to H1, thus avoiding recombination between the two haplotypes [14]. H1 is much more common than H2 and H2 expression is lower than expression of H1 [78]. H1 and H2 include several specific single nucleotide polymorphisms (SNPs) [72]. Based on H1-specific SNPs at least 23 H1 sub-haplotypes have been described [38]. Tau binds to and stabilizes microtubules in neurons. By interacting with ribosomes tau regulates mRNA translation and transports proteins in axons. It influences the function and stability of synapses [35] and is involved in neuronal differentiation and function [10]. Tau is mainly synthesized by neurons but also by oligodendrocytes and astrocytes.

**Fig. 1:**
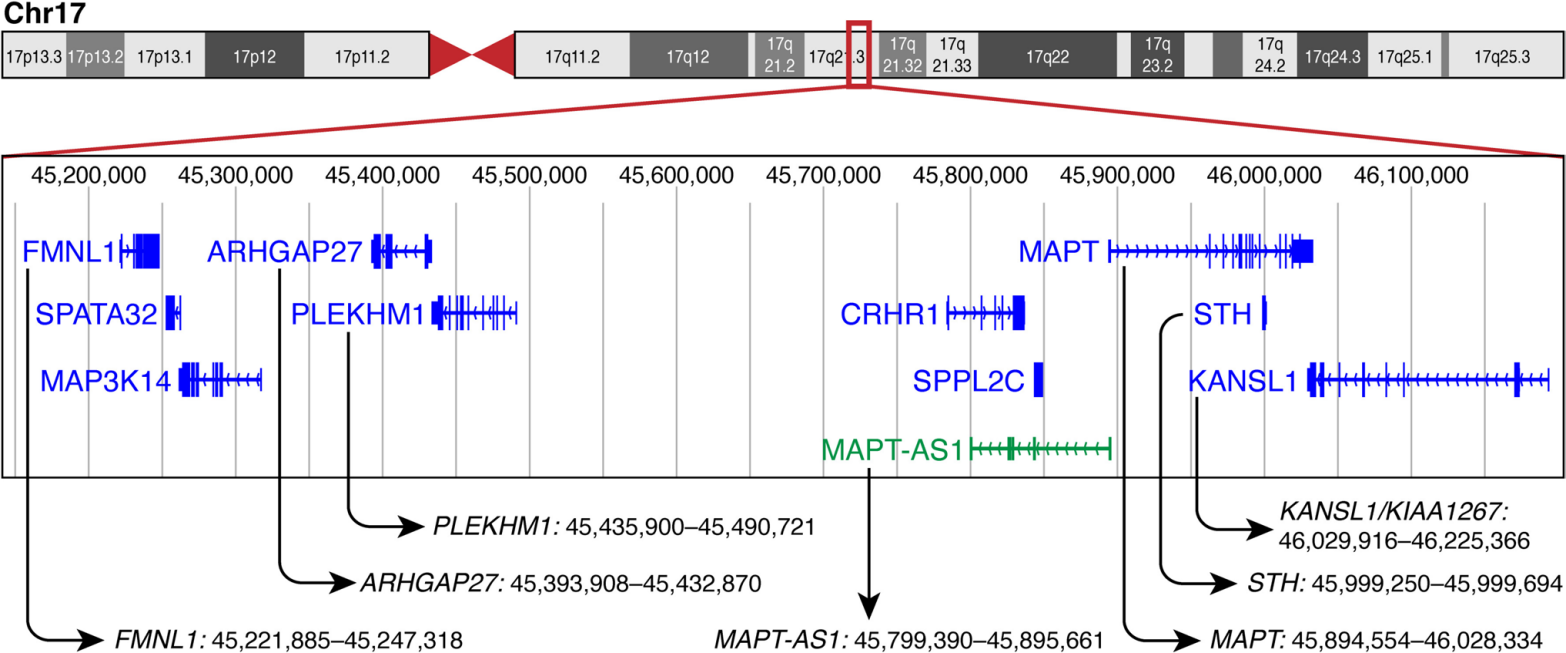
*MAPT* region in 17q21.31. Genes discussed in this article are indicated by arrows and their DNA coordinates are given. (adapted from the UCSC Genome Browser at http://genome.ucsc.edu)

The *MAPT* region (Fig. 1) extends beyond the gene proper and contains two genes (*FMNL1 and ARHGAP27*) that function as cis-regulatory elements (CREs) of *MAPT.* These CREs operate as enhancers and interact with *MAPT* promoters in neurons. There are two regions at the *FMNL1 (*Formin Like 1 with at least 3 isoforms; https://www.uniprot.org/uniprotkb/O95466/entry) locus that appear to regulate *MAPT* expression. These two regions interact with the *MAPT* promoter in both excitatory and inhibitory neurons [78]. Interestingly, expression of the *FMNL1* gene is very low in the brain. *FMNL1* obviously has two functions, one as an enhancer of *MAPT* in cells of the brain and another one as a protein coding gene in other tissues. Furthermore, there are two additional regions containing *ARHGAP27* (encoding Rho GTPas activating protein 27 with at least 4 isoforms; https://www.uniprot.org/uniprotkb/Q6ZUM4/entry), 464,677 bp and 461,949 bp upstream of *MAPT* that have enhancer function [78]. In addition, there appears to be an intronic CRE within *MAPT*. Another gene in the region, *MAPT-AS1* (*MAPT* antisense RNA 1, an RNA gene belonging to the lncRNA class) might operate as an epigenetic regulator of *MAPT* expression [19, 78].

*STH* and *KANSL1* that lie within or close to *MAPT* (figure) are co-expressed with *4RMAPT* at least in brains of Parkinson disease cases [93] suggesting interacting enhancer functions.

*MAPT* is associated with PSP risk as demonstrated by numerous findings:

1. About 15 different point mutations of *MAPT* have been detected in families with autosomal dominant inheritance of cases presenting as PSP. One of these mutations (p. L284R) is located in exon 10 [89], another one (p.E342 K) in exon11 [56] of the gene. Additional point mutations have been found in atypical PSP and FTDP-17 mainly in exon 10 of *MAPT* [8, 46]. The cases with PSP-like presentations and point mutations appear to differ from classical PSP.
2. While 3R and 4R are present in normal brains in equal amounts [29], 4R tau is preferentially found in PSP [30].
3. Of the two major haplotypes of *MAPT*, H1 is associated with increased risk for PSP and H2 is protective [31, 32]. The frequency of H1 is 95% in chromosomes of PSP patients as compared to 77.5% in control chromosomes [33, 34].
4. The H1 sub-haplotype H1c is overrepresented in PSP as compared to controls. H1c is defined by presence of an A nucleotide of the reference SNP rs242557 14. This SNP is located in the regulatory region of *MAPT* and allele A is thought to trigger higher expression of *MAPT*. This is likely to increase the amount of tau protein and thus the risk for PSP and other tauopathies such as corticobasal degeneration (CBS) [38]. Several additional sub-haplotypes of H1 (H1 d, H1 g, and H1o) are also significantly increased in PSP as compared to controls [38].
5. There are three large copy number variants (CNVs), designated α, β, and γ within H1c. While α, β do not affect the risk for PSP increased copies of γ enhance PSP risk [102].
6. Genetic association studies found highly significant association of polymorphic sequences in non-coding regions of *MAPT* with PSP, such as SNPs rs8070723 and rs242557 [43, 81]. A meta-analysis of two genome wide association studies [43, 81] yielded genome wide significance of *P* = 5.51E-144 for rs.8070723 and of *P* = 3.78E-85 for the association of PSP with rs242557. rs8070723 is located in an intron of *MAPT* [3] and rs242557 is located in a highly conserved repressor domain in the *MAPT* promoter region [6]. Tau levels are increased in all brain areas affected in PSP [44]. It accumulates in neurons, oligodendrocytes and astrocytes [29]. Up-regulation of *MAPT* contributes to the development of PSP by increasing the amount of tau and altering its posttranslational modifications (methylation, phosphorylation) and interfering with its normal functions [30]. For example elevation of tau might disturb its effect on stability of microtubules which would eventually result in cell death. In the brain the increase of tau might disturb the stability of synapses and might interfere with the function of neurons. Transcriptomic analyses showed that the increase in tau is mainly due to haplotype-dependent increased expression of *4RMAPT* [76].
7. *ARHGAP27 expression* is dysregulated in cerebellum and temporal cortex in PSP. Since dysregulated co-expression with *MAPT* was described and *MAPT* transcription is increased at least in the frontal cortex of PSP, this might indicate that expression of *ARHGAP27* is upregulated in given brain structures as well [76].

There are several genes in the *MAPT* H1/H2 inversion region such as *KANSL1, PLEKHM1* (*P* = 1.0E-9) *LRRC37A4 (P* = *2.2E-22)*, *ARL17A* (*P* = 9.2E-22) that are — as is *MAPT* (*P* = 8.71E-28) — significantly associated with SNPs within this region, in particular rs.242557. It was discussed [43] that association with markers in this region reflects proximity to *MAPT*. This is indeed the case (Fig. 1). A recent study investigated whether these genes are regulated similar to *MAPT*. Analysis of eQTLs in the H1/H2 region demonstrated that some genes in close proximity to *MAPT* are regulated independent of *MAPT* [26, 76]. Two of these genes are *KANSL1* and *PLEKHM1*.

***KANSL1 (KIAA1267)*** is located in the *MAPT* region of chromosome 17q21.31 (Fig. 1) and encodes the nuclear protein KAT8 Regulatory NSL Complex Subunit 1 (NSL1). This protein is a component of a protein complex, MLL1 and NSL1 which are involved in histone acetylation and methylation [67]. These histone modifications are probably the underlying mechanisms for their roles in mitosis, cell proliferation, and enhancer regulation [84]. Specifically, NSL1 regulates the fusion of autophagosomes and lysosomes [57]. In the mouse, reduced activity of *KANSL1* interferes with clearance of impaired mitochondria which results in an increase in reactive oxygen species and eventually in neuronal death [58]. In humans, expression of *KANSL1* is highest in the cerebellum. Among single cells in the brain expression is highest in astrocytes and lowest in vascular cells (https://v18.proteinatlas.org/ENSG00000120071-KANSL1/tissue).

Findings in RNA libraries from cerebellum of PSP patients and controls suggest differential expression of *KANSL1* and *MAPT* [26, 76]. These observations are consistent with those of a previous study [87] that also indicated independent expression of *KANSL1* and *MAPT*. Therefore *KANSL1* might convey risk for PSP independent of *MAPT*. *KANSL1* is increased in oligodendrocytes of the temporal cortex and the cerebellum of PSP in a haplotype (H1)-dependent manner [26, 76]. Although clearance of impaired mitochondria is impeded owing to a decrease in *KANSL1* activity [57], an accumulation of NSL1 might also impede autophagosome–lysosome fusion, such as the promotion of the degradation of cellular components. While *KANSL1* deficiency affects synaptic function by disturbed autophagy, increased transcription of the gene might have a similar effect [58].

***PLEKHM1*** encodes Pleckstrin Homology and RUN Domain Containing M1 (PKHM1). It is located on human chromosome 17q21.31 in the *MAPT* region (Fig. 1). PLEKHM1 is involved in the fusion of endosomes and lysosomes and in late stages of endolysosomal maturation. It may also play a regulatory role in autophagic and endocytic trafficking. Mutations in this gene have been found in patients with osteopetrosis [99]. *PLEKHM1* is expressed in most tissues. In the brain, highest expression is in the cerebral cortex and cerebellum, and medium expression in hippocampus and nucleus caudatus, and hypothalamus. Expression is highest in oligodendrocytes, followed by astrocytes, microglia, and neurons and is lowest for vascular cells (https://v18.proteinatlas.org/ENSG00000225190-PLEKHM1/tissue). PLEKHM1 interacts with Rab7 and suppresses endocytic transport [91]. In PSP *PLEKHM1* expression is increased as compared to controls [76]. This might interfere with normal function of oligodendrocytes, e.g., by causing abnormal lysosomal metabolism and/or impaired autophagosome–lysosome fusion that eventually cause death of this cell type. Significantly, several additional genes such as *MOBP, SLCO1 A1, NFASC,* and *CNTN2* (see below) are relevant for normal function of oligodendrocytes that is disturbed in PSP.

***STX6*** is located in the long arm of chromosome 1 (1q25.3) and codes for syntaxin-6. Syntaxin-6 is a SNARE (Soluble *N*-ethylmaleimide-sensitive factor attachment protein receptor) protein with various functions in cellular trafficking. It is involved in targeting of endosomal vesicles to the trans-Golgi network (TGN) and to a lesser extent from TGN to endosomal vesicles. It is also required for movement of vesicles from endosomes to the cell membrane [51, 106]. *STX6* is expressed in most tissues, yet highest in hepatocarcinoma cell lines. In the brain it is expressed in oligodendrocytes and astrocytes, neurons, microglia, and vascular cells. Expression is highest in the cerebral cortex and somewhat less in cerebellum, hippocampus, and caudate nucleus (https://www.proteinatlas.org/ENSG00000135823-STX6/tissue).

*STX6* appears to be involved in PSP. A significant association was found with an allele of SNP rs1411478 at position 180,993,146 (GRCh38 assembly) of 1q25.3 [43, 27, 81]. This SNP lies in intron 4 of *STX6* [27]. Ferrari et al. [27] found significant association (*P* = 1.8E-9) of rs1411478 with PSP. In our cohorts [43] significance of association was *P* = 2.3E-10 with the same SNP. It may be that the disease-associated allele of *STX6* influences movement of misfolded proteins from ER to lysosomes and thus may contribute to neurodegeneration. Based on specific down-regulation of *STX6* in white matter of patients as compared to controls, Ferrari et al. [27] speculated that the associated allele of *STX6* contributes to white matter pathology in PSP. This was shown on the basis that the associated risk allele rs1411478 is a strong expression quantitative trait locus. In a later study Farrell et al. (2024) [26] found that the lead SNP of their GWAS, i.e. rs1044595-C is associated with increased expression of *STX6* in brain samples and in purified oligodendrocytes [26].

***MOBP*** is located in the short arm of chromosome 3 (3p22.1) and codes for Myelin Associated Oligodendrocyte Basic Protein [65, 109]. *MOBP* is expressed in the white matter of the cerebral cortex, basal ganglia, hippocampal formation, midbrain, amygdala and hippocampus and — at very low levels of expression — in the cerebellum [109]. Expression is by far highest in oligodendrocytes and much lower in microglia and vascular cells, and hardly detectable in astrocytes. (https://www.proteinatlas.org/ENSG00000168314-MOBP/tissue#rna_expression). MOBP synthesized by oligodendrocytes plays an essential role in stabilizing the myelin sheath in the central nervous system.

*MOBP* is likely involved in PSP. SNP rs1768208 within an intron of *MOBP* was significantly (*P* = 1E-16) associated with PSP [26, 27, 43]. The risk allele results in higher expression of *MOBP* in patients as compared to controls [3]. The authors speculate that increased levels of MOBP result in neuropathology of oligodendroglia thus contributing to PSP risk. Performing eQTL analyses a more recent study [26] found decreased expression of *MOBP* in both frontal cortex and cerebellum. The different results concerning the regulation of *MOBP* expression are likely due to the two different methods applied. While upregulation was found in associations between PSP risk variants and temporal cortex levels of 20 genes within ± 100 kb [3], decreased expression was found by eQTL analysis [26].

***EIF2AK3*** is located on chromosome 2p11.2 and encodes Eukaryotic Translation Initiation Factor 2-α Kinase 3 (PERK). It is a metabolic stress-responsive kinase that phosphorylates eIF2α (α subunit of eukaryotic translation initiation factor 2). PERK halts protein synthesis when unfolded proteins accumulate in the endoplasmic reticulum (ER) (unfolded protein response, UPR), thus preventing ER stress [90, 111]. It is expressed in oligodendrocytes and to a lesser degree in astrocytes and neurons. Its highest expression is in vascular cells. Among other structures expression is highest in the pituitary gland.

We [43] found a highly significant association with PSP of an allele of rs7571971, which is located in intron 2 of *EIF2AK3* (*P* = 3.2E-13). Analyzing a different patient cohort and using a different reference SNP (rs13003510) Wang et al. [101] did not find a significant association of *EIF2AK3* with PSP. Similarly Farrell et al. [26] failed to detect such an association. Two additional studies, however, confirmed genome-wide significant association of *EIF2AK3* with PSP [81, 90]. The UPR is activated/impaired in PSP probably by reduced PERK expression and function [111] thus worsening tau accumulation in PSP [90].Yuan et al. [111] propose that the reduction of PERK function results in vulnerability to ER stress and damage that increases neurodegeneration. Consistent with these findings is the observation that activation of PERK attenuates tau pathology in vivo and in vitro [13]. These findings suggest that *EIF2AK3* expression is reduced in PSP. Insufficient amounts of PERK might contribute to neurodegeneration.

***SLCO1A2*** is located on chromosome 12p12.1. It encodes Organic Anion-Transporting Polypeptide 1A2 (OATP1A2). *SLCO1A2* is expressed at equal amounts in various tissues including all structures of the brain but among single cells of the brain it is almost exclusively expressed in oligodendrocytes. The major functions of OATP1A2 are mediation of cellular uptake of organic ions such as steroidal compounds and the uptake of various drugs, thus influencing their metabolism, distribution and excretion [36, 55].

Meta-analysis of two genome-wide association studies [43, 81] showed significant association of PSP (*P* = 5.3E-10) with an allele of SNP rs11568563 located in exon 5 of *SLCO1A2* [115]. The base change of this allele gives rise to the non-synonymous p.E172D mutation in the protein. Association of this SNP with PSP is likely to be mediated by this mutation. pE172D affects a region of the 4^th^ trans-membrane domain of the anion-transporting polypeptide. This might interfere with the normal function of OATP1A2 and result in decreased expression of *SLCO1A2* in the brain of PSP patients [117]. Lower expression as found in PSP appears to result in decreased uptake and excretion of metabolic toxic substances from the brain thus contributing to the risk of PSP. Another investigation confirmed a decreased expression of *SLCO1A2* in the frontal cortex and cerebellum [26].

***DUSP10*** codes for Dual Specificity Protein Phosphatase 10 (DUSP10) and is located on chromosome 1q41. The gene is expressed in most brain structures, in oligodendrocytes and microglia, and at a very low level in vascular cells. Main functions of *DUSP10* include negative regulation of members of the mitogen activated protein (MAP) kinase superfamily. As important regulators of MAP-kinase signaling dual-specificity protein phosphatases (DUSPs) play an important role in neuronal differentiation, synaptic plasticity and survival [69].

*DUSP10* is significantly associated with PSP. Meta-analysis of two genome wide association studies [43, 81] detected significant association of an allele of SNP rs6687758 with *DUSP10* (*P* = 1.14E-08) [105]. This SNP is located in an intergenic region between *DUSP10* and *TRT-TGT2-1*. Although *DUSP10* is found in brain regions affected in PSP, its contribution to PSP development is not quite clear. It is possible, however, that the associated allele interferes with some neuronal functions, which might result in abnormal tau phosphorylation, compromised synapse operation and glial pathology [69]. Presently it is not known whether *DUSP10* is up- or down-regulated in PSP in some structures of the brain.

***APOE*** is located on chromosome 19q13.32 and encodes apolipoprotein E (APOE). Highest expression is found in liver and brain. Expression levels in basal ganglia, midbrain, and pons are somewhat higher than in other brain structures. Expression of *APOE* is high in astrocytes, and much lower in microglia and vascular cells. (https://www.proteinatlas.org/ENSG00000130203-APOE/brain). There are three major alleles that differ at two amino acid positions in the protein (positions 112 and 158). *APOE-ε2* encodes APOE with amino acid cysteine at positions 112 and 158 (cys112, cys158), APOE variant *ε3* is characterized by cys112 and arg158 and variant *ε4 by* arg112, arg158. APOE is mainly secreted by the liver but some additional tissues/organs secrete this protein as well. APOE plays a major role in the lipoprotein-mediated transport of lipids. It binds to cell surface receptors and to lipoprotein particles. Functions such as the transport of lipids are reduced in APOE ε2 as compared to APOE ε4. In the brain APOE is secreted by astrocytes and regulates the metabolism of lipids, such as the transport of cholesterol and phospholipids from glia to neurons [70, 71].

Whole genome sequencing identified highly significant association between *APOE-ε2* (characterized by rs429358-T and rs7412-T) and PSP (*P* = 9.57E-16) [101] thus confirming a previous study in a relatively small cohort of Japanese PSP patients [82]. *APOE-ε4* decreases and *ε2* increases risk for PSP. The opposite is true for Alzheimer disease (AD) where *APOE-ε2* is protective and *ε4* is a major risk factor [114]. The reasons are unclear. Perhaps APOE ε2 and APOE ε4 act differently in the presence of either 4R-tau only (as in PSP) or both 4R and 3R tau as in AD (3R-4R-tau). Differences in aggregation between 4R-tau and 3R-tau might also contribute to the different responses to APOE ε4 and APOE ε2 in PSP and AD. This suggestion is supported by studies of Siddiqua and Margittai [86]. While filaments of 4R tau or 3R tau only are highly ordered, mixed 3R tau and 4R tau assemble to form heterogeneous filaments thus demonstrating that 4R-tau filaments in PSP are different from the 3R-4R filaments found in AD. Presently, it is not known whether the expression of *APOE* is altered in PSP.

(Wang et al. [101] also demonstrated the usefulness of identification of multiple rare variants (SVs SNVs, indels etc.) within and/or around a given gene for the analysis of their potential association with a disorder. These analyses are referred to as genetic burden tests. Significance of a disease burden is determined by the frequency of rare associated variants in a given gene in controls vs. disease. Wang et al. identified 16 rare variants (one affecting a splice donor site the other 15 damaging missense variants) within the gene *ZNF592* and determined a burden significance of *P* = 7.3E-06. *ZNF592* is located on chromosome 15q25.3 and encodes zinc finger protein 592. Among other processes it is required for normal cerebellar development.

***RUNX2*** encodes the nuclear protein RUNX family transcription factor 2. The gene is located on chromosome 6p21.1. Its main function involves regulation of osteogenesis and skeletal morphology. In the human and mouse brain it is mainly expressed in the cortex, cerebellum, putamen, substantia nigra, suprachiasmatic and paraventricular nuclei and hippocampus [15, 26]. According to the human protein atlas *RUNX2* is almost equally expressed in most structures of the human brain but lowest in cerebellum. In the glia, expression in astrocytes is higher than in oligodendrocytes, however, expression is much lower than in microglia and vascular cells (https://v22.proteinatlas.org/ENSG00000124813-RUNX2/brain). *RUNX2* plays an important role in in the regulation of circadian and diurnal rhythms as shown in the mouse [75]. It is also involved in neuronal development and growth [12, 103] as well as regulation of neuronal regeneration [45].

Significant association with PSP was found in an allele of SNP rs35740963 (chr. 6 nucleotide position 45,531,877) (*P* = 1.8E-8). This SNP is located within *RUNX2* (chr. 6 positions 45,328,157–45,664,349) [15]. A highly significant association (*P* = 5.43E-12) with *RUNX2* was confirmed for an intragenic SNP locus rs12197948 (position 45,454,844) [26]. Expression of *RUNX2* is increased in PSP as shown by the association of rs12197948-A with increased *RUNX2* expression. The closely associated reference SNPs rs12197948 and rs4714854 are located within intron 3 of *RUNX2*, a region of the gene that contains an enhancer that is active in both microglia and oligodendrocytes. This suggests that dysregulated *RUNX2* has an effect on normal microglial function. Dysfunctional microglia compromise many cellular processes such as protection of immune defense, maintenance of homeostasis of the CNS, and shaping of neuronal synapses, as well as protection against neuroinflammation. Disturbance of these functions can increase tau burden, cause neuroinflammation and eventually neuronal death [4, 18, 31, 47].

***TRIM11*** is located on chromosome 1q42.13. It encodes Tripartite Motif containing Protein 11. *TRIM11* is expressed mainly in neurons and in all brain structures. Expression is highest in the cerebellum, but it is also high in basal ganglia and cerebral cortex, compared to other brain regions. It is expressed in oligodendrocytes, but higher in neurons and vascular cells (https://v22.proteinatlas.org/ENSG00000154370-TRIM11/tissue). TRIM11 has an E3 ubiquitin ligase activity and it mediates degradation of various proteins such as tau fibrils and excessive normal tau and interferes with generation of protein aggregates [116]. TRIM11 protects against tauopathies [113]. It plays a role in neuronal function via regulation of the neurogenic transcription factor Pax6 [96].

*TRIM11* is significantly associated with an allele of rs564309 (*P* = 1.7E-9) in a cohort of PSP patients composed of classical Richardson syndrome (RS) and non-RS [50]. rs564309 is located in intron 3 of *TRIM11*. A follow-up study showed that the minor allele, rs564309-A, is associated with increased neurofibrillary tangles in PSP but is not significantly associated with other tau pathology scores such as neuropil threads, coiled bodies, or tufted astrocytes. This allele also does not affect age in PSP [98]. The expression level of *TRIM11* in PSP is not known but it has been speculated to vary between brain regions and to differ between classical PSP and atypical PSP. *TRIM11*, however, has been shown to be downregulated in the brains of AD patients resulting in reduced degradation of proteins such as tau. Since both AD and PSP are tauopathies, a similar mechanism might apply to PSP and therefore *TRIM11* is expected to be downregulated in PSP brains [113].

***NFASC*** and ***CNTN2*** are located on chromosome 1q32.1. Both genes are approximately 20 kb apart with *CNTN2* being located downstream of *NFASC*. *NFASC* and *CNTN2* play important, partially complementary roles in the nervous system. *NFASC* is highly expressed in neurons and oligodendrocytes. It encodes neurofascin which is required for the formation of paranodal junctions at the Ranvier nodes, myelination and conduction of nerve impulses in both the central and peripheral nervous system [22, 32, 53]. *CNTN2* codes for contactin-2, a cell adhesion molecule required for the formation of paranodal junction at the nodes of Ranvier. It is almost exclusively expressed in oligodendrocytes, supports the function of this cell type, maintains voltage-gated potassium channels and is involved in myelination [63]. Both genes are treated as one locus due to their close proximity to each other, related functions in the nervous system, and identification with one SNP in association studies (see below).

A meta-analysis including a cohort of previously not studied PSP cases from Spain and Portugal and previously analyzed cases identified a signal at 1q32.1. This signal (rs12744678) showed highly significant (*P* = 4.15E-8) association with *NFASC* [39]. Additional analyses, mentioned in this article but not published, showed that the SNP, rs4951151, is an eQTL for *CNTN2* in cerebellar tissue and a pQTL for *CNTN2* in CSF and plasma. Taken together these association studies reveal the biologic functions of the *NFASC*/*CNTN2* locus that are related to myelination and axon ensheathment. Significantly, other genes associated with PSP such as *MOBP* and *SLCO1 A1* (see above) are co-expressed with *NFASC*/*CNTN2* and also play major roles in oligodendrocyte-specific myelination.

***LRRK2*** encodes leucine-rich repeat kinase 2 also referred to as dardarin or PARK8. *LRRK2* is located on chromosome 12q12. The protein has several different domains: A leucine-rich repeat (LRR) domain, a kinase (K) and GTPase domain, protein–protein interaction domains (armadillo (ARM), ankyrin (ANK), and WD40 domains) that are relevant for protein–protein interactions. Major cellular functions of dardarin include phosphorylation of tubulin, tau and moesin, that is important for maintenance of tubular and cytoskeletal integrity; mediation of vesicle and membrane trafficking; and — in the nervous system — the regulation of synaptic function, and the outgrowth of dendrites and axons. LRRK2 also affects G-protein signaling via its kinase activity. The gene is expressed in multiple human tissues including brain where it is expressed in neurons, oligodendroglia, astrocytes and microglia [33, 40, 64, 92, 112].

While GWAS have not identified *LRRK2* as a risk factor in PSP, there is evidence for this gene as a modifier of survival in PSP. A highly significant association between rs2242367 and PSP survival (*P* = 7.5E-10) was shown by the study of 1001 pathology-confirmed cases of white European ancestry. Analysis of a second cohort revealed a trend toward an association of rs2243357 with PSP survival but these findings were not statistically significant. However, a combined analysis of both cohorts (*n* = 1239) gave highly significant association of rs2242367 with survival in PSP (*P* = 1.3E-10). The minor allele of rs2242367 was associated with worsening survival [49]. rs2242367 was also associated with the long noncoding RNAs *LINC02555* and *AC079630.4*. Interestingly, the minor allele of rs2242367 that was associated with reduced survival resulted in an increase in the expression of *LRRK2*, *LINC02555*, and *AC079630.4*. This association of rs2242367 with both *LINC02555* and *LRRK2* suggests that *LINC02555* controls *LRRK2* expression in specific cell types of the brain. This assumption is supported by findings that some lncRNAs control the expression of *LRRK2* [23].

Potential modes of increased *LRRK2* expression in PSP can be derived from the physiologic functions of *LRRK2* and findings in Parkinson disease where *LRRK2* is an important risk factor. Thus pathogenic mutations in *LRRK2* result in phosphorylation of some Rab proteins which play an important role in trafficking of intracellular vesicles [2, 49] which in turn results in disturbed proteostasis and induction of inflammation [61]. Mutations in *LRRK2* have also been shown to be associated with tau pathology. Thus *LRRK2*-regulated endocytosis leads to extracellular tau uptake by neurons [24, 49]. These findings demonstrate a role of pathologic tau-uptake by neuronal cells and abnormal trafficking of intracellular vesicles. Upregulation of *LRRK2* might have the same or similar effects as *LRRK2* mutations thus resulting in incremental disturbance of neuronal cell function which in turn might affect the survival in PSP. Based on the findings of a role of *LRRK2* in the regulation of synaptic function and outgrowth of dendrites and axons it is also possible that increased *LRRK2* expression interferes with these processes that might result in slowly increasing neuronal cell death that eventually results in PSP with variable survival.

### Additional possible PSP risk genes

Another risk gene for PSP was suggested by experiments that went beyond common GWAS studies since this gene (possibly *C4A*) had to be identified in a gene-dense region of the human genome, the RCCX locus and surrounding areas within the HLA class III region on 6p21.33 (https://www.genecards.org/cgi-bin/carddisp.pl?gene=WHR1).

***C4A*** codes for the acidic form of complement factor 4. It is located at the RCCX locus within the MHC class III region on chromosome 6p21.33. This highly complex multiallelic locus is composed of 1-4 tandemly repeated modules, long and short. The long module includes genes *STK19* (partial)-*C4A*-*CYP21A1P* (pseudogene)-*TNXA*, the short module harbors *STK19*-*C4B*-*CYP21A2*- partial *TNXB*. The proteins encoded by these genes have endocrine (e.g., in the synthesis of some steroid hormones), connective tissue (e.g., synthesis of glycoproteins) and immune (e.g., involvement in complement-mediated immune response) functions [85]. Probably owing to its involvement in some functions of the immune system, the region is implicated in neurodevelopmental and neuropsychiatric disorders, including schizophrenia and chronic fatigue syndromes. Apart from its role in the innate immune system, *C4A* is also involved in synaptic pruning that is mainly active during infancy up to puberty but to some degree also operates during adult life [107].

Performing a GWAS in a large cohort of autopsy-confirmed PSP cases and controls Farrell et al. [26] replicated *RUNX1,* located within 6p21.1 as a risk gene for PSP (see above). They also identified a novel locus on 6p21.33 near *TNXB* at SNP rs369580 (P = 8.11E-10). Given the gene density within 6p21.32–6p21.33 additional experiments had to be performed to identify a potential PSP risk gene within this region. Applying the program INFERNO [5] they identified three sets of SNPs. Co-localization of each SNP set with nearby genes was tested using eQTLs from 13 brain regions. Co-localization was highest for *C4A* expression. Immunohistochemical staining for C4A revealed a significantly higher number of “C4A positive axons in association with p-tau positive oligodendrocytic coiled bodies” in PSP as compared to controls. These and additional experiments performed by the authors suggest that *C4A* located within the long module of RCCX is a risk gene for PSP. Although the results are intriguing, further studies are needed to exclude the possibility that different gene(s) within RCCX and surrounding regions convey risk for PSP.

***IRF4*** encodes Interferon Regulatory Factor 4, which plays a decisive role in various stages of B-cell development. *IRF4* is located on chromosome 6p25.3.

In an earlier study we found highly significant association of *IRF4* SNP rs12203592 with PSP (*P* = 6.3E-15) [43]. However, the allele frequencies of rs12203592 reported in older controls differed significantly from our controls. The same difference in allele frequency was found for the adjacent SNP rs2493013. We therefore did not consider a putative association with *IRF4* reliable. In 2018 Sanchez-Contreras et al. [81] performed a meta-analysis of association of *IRF4* SNP rs12203592 with PSP. This study included our previous dataset. Since the result was solely driven by our initial findings (the added cohort did not contribute to the association), an age-related bias of rs12203592 frequencies in controls may have distorted the association findings in this meta-analysis as well. As the authors point out, additional studies are required to solve the problem of a possible association of *IRF4* with PSP.

## Synopsis

The 14 genes shown to be highly significantly associated with increased risk for PSP in association studies are expressed in all brain structures as listed in the Human Protein Atlas (https://www.proteinatlas.org). At the cellular level most of these genes are transcribed in oligodendrocytes, astrocytes and neurons, but others are highly expressed in microglia and vascular cells which are important for normal brain function as well.

The likely consequences of dysregulation of each of the fourteen PSP risk genes have been discussed above. The following mainly addresses the possible interplay of these genes and their products. It also investigates potential related functions of these genes.

Presently no physical interaction is known between the proteins encoded by these genes and tau, the major protein that is altered in tauopathies. Yet tau interacts with a wealth of proteins involved in the regulation of cytoskeleton, association with synaptosomal proteins [94], microtubular proteins [21, 104, 108], and with kinases and phosphatases [62, 66]. In fact, *LRRK2* indirectly regulates tau (probably mainly microtubule-associated tau) phosphorylation via the glycogen synthase kinase 3β [37, 52]. Furthermore, some other genes dysregulated in PSP might also act indirectly (downstream) of the known tau interacting proteins. This may be the case for *DUSP10*. DUSP10 regulates MAP kinases, that in turn regulate phosphorylation of tau. Thus, similar to *LRRK2*, altered expression of *DUSP10* in PSP might affect the function of tau via abnormal phosphorylation that eventually causes neuronal death. *NFASC*, *CNTN2*, *SLCO1A2*, and *MOBP* are co-expressed and their abnormal (upregulated) expression might interfere with oligodendrocyte-mediated myelination and ultimately result in neuronal death.

Several of the 14 genes associated with PSP increase disease risk by common mechanisms. *KANSL1* regulates autophagosome-lysosome fusion. This is an important step in the maturation of phagosomes [57]. Mature phagosomes are required for acquisition of microbicidal properties for the correct function of the innate immune response [25]. *PLEKHM1* regulates the fusion of endosomes and lysosomes and is required for the maturation of endolysosomes. Endolysosomes are involved in the degradation of macromolecules. In neurons endolysosomes are involved in clearance of synaptic vesicles and axons by degradation of misfolded proteins [25, 48, 79]. The UPR is compromised by decreased expression of *EIF2AK3* [41, 74, 90]. *STX6* codes for syntaxin-6, which localizes to endosomes and the TGN. The Leucine-rich repeat kinase2 encoded by *LRRK2* physically interacts with syntaxin-6 at the TGN. Specifically, an interaction occurs between the SNARE proteins syntaxin-6 and VAMP4 and GARP (Golgi-associated retrograde protein) that facilitates efficient fusion of retrograde transport vesicles with TGN [11]. This is a prerequisite for efficient retrograde trafficking, i.e., the transport of proteins and lipids, from the Golgi apparatus toward the ER (endoplasmic reticulum) [88]. *TRIM11* is required for degradation of various proteins including tau. Taken together, malfunction of these six genes compromises vesicle/membrane fusions, interferes with the UPR and increases ER stress. The six genes compromise clearance of misfolded proteins either independently or in concert.

An additional two genes might cause neuronal death by interference with the movement of various compounds either between cells (*APOE*) or from extracellular space into cells (*SLCO1A2*).

The discovery of these risk genes and their potential pathologic mechanisms in PSP now facilitates experimental studies into their normal and disturbed function. A detailed understanding of the mode of action of these genes — and perhaps of additional ones that have not yet been discovered yet — will make possible the development of disease-modifying therapies for this disabling neurodegenerative disease.

## Data Availability

No datasets were generated or analysed during the current study.
